# Toxicity of Ammonia Nitrogen to Ciliated Protozoa *Stentor coeruleus* and *Coleps hirtus* Isolated from Activated Sludge of Wastewater Treatment Plants

**DOI:** 10.1007/s00128-012-0816-3

**Published:** 2012-09-14

**Authors:** Beata Klimek, Janusz Fyda, Agnieszka Pajdak-Stós, Wioleta Kocerba, Edyta Fiałkowska, Mateusz Sobczyk

**Affiliations:** Institute of Environmental Sciences, Jagiellonian University, Gronostajowa 7, 30-387 Kraków, Poland

**Keywords:** Activated sludge, Ciliated protozoa, NH^4+^ ion, Ammonia toxicity

## Abstract

We assessed the toxicity of ammonia ions to *Stentor coeruleus* and *Coleps hirtus* (Protozoa) isolated from activated sludge taken from two municipal wastewater treatment plants in southern Poland. *Stentor coeruleus* is a rarely occurring species in activated sludge, unlike the widespread *Coleps hirtus*. The mean LC50 values (concentration causing 50 % mortality) calculated for the 24 h tests differed hugely between the tested species: 43.03 mg NH^4+^ dm^−3^ for *Stentor coeruleus* and 441.12 mg NH^4+^ dm^−3^ for *Coleps hirtus*. The ammonia ion concentration apparently is an important factor in the occurrence of these protozoan species in activated sludge.

Human activities have increased the nitrogen concentrations in water worldwide, leading to considerable eutrophication (Bressan-Buosi et al. 2011). Ammonia nitrogen is a very common chemical form in aquatic ecosystems, and its toxic effects have been widely reported (Ankeley et al. 1995; Monda et al. 1995), including effects on marine ciliates (Xu et al. 2004). Ammonia is a common pollutant in wastewater and may be present in either non-ionized and ionized forms. Non-ionized ammonia can pass through cell membranes and harm cells due to its fat solubility. Ammonia can reach high concentrations in wastewater, with detrimental effects on activated sludge used in wastewater treatment systems. Ammonia nitrogen concentrations have been reported as high as 162 mg dm^−3^ in domestic wastewater (Drzewicki and Kulikowska 2011) and 4,950 mg dm^−3^ in industrial wastewater (Zhang et al. 2012).

Many free-living protozoan species are distributed worldwide in various types of habitats (Finlay 1998; Bamforth et al. 2005; Fyda et al. 2006; Bartošová and Tirjaková 2008). Protozoa are one of the most important grazers of microbes in aquatic environments and the only grazers of any importance in anoxic habitats (Finlay and Esteban 1998). As the problem of clean water availability has come to the fore, effective wastewater purification systems for industrial and municipal sludge have been developed in many countries. Activated sludge treatment, the biological treatment process most widely used for both domestic and industrial wastewater in the developed world, is an aerated oxidation process (Moo-Young and Chisti 1994; Burgess et al. 1999). Activated sludge plants have been used to treat a wide range of waste types, using chemical, biological and physical agents to effectively accelerate natural processes.

Activated sludge is a specific nutrient-rich environment inhabited by several species of bacteria, protozoa, fungi and small animal organisms. Microscopy of activated sludge to identify the major organism groups yields information about the quality of the sludge purification process, and may reveal the presence of toxic substances in wastewater (Madoni 1994; Madoni et al. 1996). Al-Shahwani and Horan (1991) showed that wastewater treatment system performance can be predicted on the basis of one of these groups, protozoa. Protozoa, especially ciliates, are generally the predominant taxa when activated sludge performs well (Chen et al. 2004). According to Finlay and Esteban (1998), ciliated protozoan species can be divided into three basic ecological groups: raptorial, catching relatively large food items; true filter feeders, using a filter to remove microbial food from suspension; and diffusion feeders, which capture swimming prey colliding with their sticky tentacles, through which the contents of prey are sucked. Both *Stentor coeruleus* and *Coleps hirtus* species are true filter feeders. They do not occur to the same extent in activated sludge. *Coleps hirtus* is widespread in activated sludge and is considered relatively tolerant, while *Stentor coeruleus* is scarce and probably more susceptible to negative environmental conditions (Martín-Cereceda et al. 1996).

Madoni’s sludge biotic index (SBI) has been developed to display the results of microscopic analysis of activated sludge and the numerical values for the quality of sludge and superb summarized the knowledge on the ecology of activated sludge (Madoni 1994; Madoni et al. 1996). However, SBI has drawn some criticism recently: sudden shock loadings of highly concentrated wastewater or the addition of a toxic influent, especially in cases of co-treatment of municipal and industrial wastewater, may result in high (desirable) SBI values even when effluent quality is low (Papadimitriou et al. 2007; Drzewicki and Kulikowska 2011). A change in the abundance of selected protozoan species may be a more sensitive indicator of effluent quality than SBI (Drzewicki and Kulikowska 2011). Although the usefulness of the SBI in other cases is obvious, the example of shock loadings makes it clear that we need to know the tolerance of particular species inhabiting activated sludge to the range of compounds occurring in wastewater.

Protozoan species vary in their susceptibility to ammonia nitrogen, and acclimation may make protozoa more tolerant to ammonia nitrogen toxicity (Puigagut et al. 2005). Here we assess the harmful effect of ammonia nitrogen on two protozoan species, *Stentor coeruleus* and *Coleps hirtus*. We measured the 24 h acute toxicity of ammonia ions in order to evaluate the LC50 values, that is, the concentrations causing 50 % mortality in 24 h tests. To the best of our knowledge there are no such data available for these species.

## Materials and Methods

Clonal populations of *Stentor coeruleus* and *Coleps hirtus* were obtained from single individuals isolated from activated sludge taken from two different treatment plants in southern Poland. Under a magnifying glass, single individuals were transferred with a micropipette from a sludge sample to a separate Petri dish filled with Żywiec brand mineral water (free of ammonia nitrogen). *Stentor coeruleus* was fed on bacteria growing on a single wheat grain per dish, changed if needed. *Coleps hirtus* was fed with pieces of freshly killed and cut sludge worm (Oligochaeta). Both species were cultured in darkness at 20°C (Sanyo Versatile Environmental Test Chambers). The cultures were maintained continuously in the laboratory for approximately 1 year.

To assess ammonia nitrogen toxicity we performed pilot tests with a logarithmic series of ammonia nitrogen concentrations, ranging from 10,000 to 0.1 mg dm^−3^, plus control treatments. The solutions were prepared with ammonium chloride (NH_4_Cl) dissolved in Żywiec brand mineral water. Ten individual protozoa were micropipetted and transferred to separate wells (24-well Cell Wells™, Corning), with care taken to transfer the smallest possible volume of liquid with the tested protozoan cells. Then 1 ml of a given solution was added to each well. The culture plates were incubated at 20°C and the number of live and dead protozoa was checked after 24 h exposure. The pilot experiment employed three replicates of each concentration.

The lowest concentration with 100 % mortality was taken as the upper limit and additional intermediate concentrations were included in the final test. Four concentrations of ammonia nitrogen were applied for *Stentor coeruleus* (100, 50, 10, 1 mg dm^−3^, plus control), and three for *Coleps hirtus* (1,000, 500, 100 mg dm^−3^, plus control). The main experiment used ten replicates for each species and concentration. Tests in which mortality in the control was 5 % or higher were considered invalid.

The actual ammonia nitrogen concentrations in the tested solutions were analyzed simultaneously with an FIA compact analyzer with autosampler (MLE GmbH Dresden, Germany). The LC50 values (mg dm^−3^) were calculated from a linear model in Statgraphics Centurion (StatPoint Technologies Inc., Warrenton VA, U.S.A.).

## Results and Discussion

The LC50 value for ammonia ions determined for *Stentor coeruleus* was 43.03 mg dm^−3^. The LC50 value for *Coleps hirtus* was much higher at 441.12 mg dm^−3^. Table 1 gives the confidence intervals and R^2^ values for these assessments.

**Table 1:** Toxicity of ammonia ions to selected protozoa species

Species	LC50 value (mg dm^−3^)	R_adj_^2^ (%)	95 % confidence interval (mg dm^−3^)	
			Lower limit	Upper limit
*Stentor coeruleus*	43.03	49.05	38.44	47.61
*Coleps hirtus*	441.12	79.77	407.98	474.27

The tested species differed greatly in their ammonia nitrogen tolerance. The ammonia concentration evidently is an important factor affecting the occurrence of the tested species in activated sludge. As mentioned, domestic wastewater can contain ammonia nitrogen concentrations much higher than the tolerance level we established here for the more sensitive *Stentor coeruleus* (Drzewicki and Kulikowska 2011; Zhang et al. 2012).

Martín-Cereceda et al. (1996) analyzed sludge samples from several wastewater treatment plants and found *Coleps hirtus* relatively abundant. Bonnet et al. (1996) described *Coleps hirtus* as dominant among other protozoan species. Chen et al. (2004) also found *Coleps hirtus* to be relatively frequent in activated sludge. Burgess et al. (1999) categorized *Coleps hirtus* as a species associated with low concentrations of ammonia nitrogen, but our data confirm its high tolerance of ammonia.

Here we demonstrated that *Stentor coeruleus* is more sensitive to ammonia concentrations than *Coleps hirtus*. Neither Martín-Cereceda et al. (1996) nor Chen et al. (2004) found *Stentor coeruleus* in the sludge samples they analyzed. *Stentor coeruleus* as well as other *Stentor* taxa are rarely reported in activated sludge; they are found rather in permanent ponds and other natural water bodies, but even in the natural environment *Stentor coeruleus* was less frequent than *Coleps hirtus* (Kunsch 1998; Xu et al. 2005; Bressan-Buosi et al. 2011). In a study of lake protozoa, Xu et al. (2005) observed that almost all taxa of large-bodied ciliates such as *Euplotes*, *Stentor*, *Paramecium* and *Stylonychia* frequently found in samples taken in other seasons drastically declined in abundance in the summer, progressively replaced by small-bodied ciliates such as *Cyclidium*, *Trochilia*, *Cinetochilum*, *Halteria* and *Aspidisca*. By way of explanation they stated that summer cyanobacterial blooms in eutrophic waters produce a variety of toxic, bioactive secondary metabolites which clearly affect the community structure of protozoa. However, protozoa may persist locally for long periods in a cryptic state (Finlay 1998), so a population may recover after high loads of ammonia nitrogen or toxins.
